# Educational Impact of a 30-Minute Ultrasound Training Lecture on Medial Meniscus Extrusion Measurements: A Pilot Study of Intra- and Inter-rater Reliability Among Novice Students

**DOI:** 10.7759/cureus.109235

**Published:** 2026-05-19

**Authors:** Ryosuke Tozawa, Narumi Ishii, Yusuke Minamoto

**Affiliations:** 1 Department of Physical Therapy, Faculty of Health Science, SBC Tokyo Medical University, Urayasu, JPN; 2 Department of Rehabilitation, Health Science University, Fujikawaguchiko, JPN

**Keywords:** inter-rater reliability, intra-rater reliability, medial meniscus extrusion, novice, physical therapist, student, training, ultrasonography

## Abstract

Background: Medial meniscus extrusion (MME) is a pivotal biomarker for knee osteoarthritis progression. Although ultrasonography offers a dynamic and cost-effective MME assessment, its clinical utility is often limited by the perceived high barrier of examiner proficiency. This study evaluated the educational impact of a brief, standardized training session on the reliability of MME measurements obtained by novice physical therapy students compared to those obtained by an experienced physical therapist (PT).

Methods: The participants in this pilot study comprised eight healthy young adults (16 knees), one experienced PT (eight years of experience), and two third-year students (Students A and B) without prior MME measurement experience. The students attended a targeted 30-minute lecture on knee sonoanatomy and MME measurement protocols. MME was measured under weight-bearing (WB) and non-WB (NWB) conditions using a longitudinal scan directly beneath the medial collateral ligament. Reliability was assessed via intraclass correlation coefficients (ICC(1, 1) and ICC(2, 1)) and a Bland-Altman analysis.

Results: Following the 30-minute session, both students achieved moderate intra-rater reliability (ICC(1, 1): 0.693-0.705). Inter-rater agreement with the PT was highly inconsistent across conditions and individuals; for the WB condition, Student B demonstrated excellent agreement (ICC(2, 1): 0.895), whereas Student A demonstrated poor agreement (0.204). For the NWB condition, Student A showed moderate agreement (ICC(2, 1): 0.589), whereas Student B showed poor agreement (0.220). The Bland-Altman analysis identified a proportional bias only for Student A’s intra-rater measurements (between the second and third trials) under the NWB condition. Random errors (minimal detectable change at 95% confidence level (MDC95)) were 0.34-0.50 mm for student intra-rater trials and 0.40-0.65 mm for inter-rater comparisons.

Conclusions: A 30-minute educational intervention enabled novice physical therapy students to occasionally achieve expert-level reliability for specific conditions; however, it did not ensure consistent proficiency across different physical settings. The significant variability between the students and the presence of proportional bias in NWB measurements suggest that theoretical knowledge alone is insufficient to master the technical nuances of MME ultrasonography. These findings highlight the potential for ultrasound in clinical education while emphasizing the necessity of intensive hands-on training and feedback to bridge the gap between novice and expert performance.

## Introduction

Medial meniscus extrusion (MME) is the displacement of the medial meniscus beyond the tibial plateau margin [1-3]. MME significantly impairs the load-distribution function of the meniscus and is recognized as a primary factor in the progression of knee osteoarthritis because it increases stress on articular cartilage [1,2,4,5]. In recent years, ultrasonography has been increasingly utilized alongside conventional magnetic resonance imaging for MME evaluation [6,7]. Ultrasound is not only portable and non-invasive but also uniquely capable of weight-bearing (WB) and dynamic assessments [8]. Previous studies have reported that MME increases significantly under WB compared to non-WB (NWB) conditions [6,8,9], which holds substantial clinical value for diagnosing early-stage knee osteoarthritis and identifying pain sources [8]. Although prior research has demonstrated excellent reliability for ultrasound-based MME measurement (intra-rater intraclass correlation coefficient (ICC): 0.942, inter-rater ICC: 0.904), these results were generally obtained by highly experienced examiners.

Ultrasonographic evaluation is very dependent on the technical proficiency and anatomical knowledge of the examiner [10]. Consequently, several studies have investigated the impact of examiner experience on the reliability of MME measurements [11,12]. Cho et al. [12] demonstrated that 97% of measurements obtained by examiners with one and 10 years of experience fell within a 2.0 mm error range. However, that study involved chiropractors with existing medical and anatomical expertise; thus, it remains unclear to what extent individuals without prior MME-specific training can achieve diagnostic accuracy following a brief educational session. Clarifying this is essential for developing guidelines for ultrasound integration in clinical education.

Therefore, this study evaluated the educational impact of a brief, standardized training program on the proficiency for measuring MME among physical therapy students without prior MME-specific ultrasound training. Specifically, we investigated whether a 30-minute targeted lecture on sonoanatomy and measurement protocols would enable novices to achieve acceptable measurement reliability comparable to that of an experienced physical therapist (PT) under both WB and NWB conditions. By analyzing measurement consistency and identifying potential technical hurdles such as anisotropy, this study sought to provide insights regarding the feasibility and limitations of rapid ultrasound skill acquisition in clinical education.

## Materials and methods

The participants for the knee assessments were eight healthy young adults (four men and four women). Their mean height (standard deviation (SD)) was 160 (4.4) cm, mean weight (SD) was 55 (5.0) kg, and mean BMI (SD) was 20.1 (1.0) kg/m². Those with a history of knee surgery or current knee pain were excluded. The examiner participants comprised one PT with eight years of clinical experience (including >3 years of ultrasound experience) and two third-year physical therapy students (Students A and B). The students had completed 180 minutes of didactic lectures and 60 minutes of hands-on practice regarding ultrasonography, although they had no prior experience with MME measurements. The students possessed knowledge of systemic anatomy; however, they had not received specific training in knee sonoanatomy. Prior to the study, the students attended a 30-minute educational session on MME measurement and knee sonoanatomy delivered by an independent PT with more than five years of ultrasound experience and 15 years of clinical experience. The session consisted of a didactic explanation of knee sonoanatomy and MME measurement procedures, followed by a live demonstration of probe positioning and image acquisition. The educational content included identification of medial knee sonoanatomy, probe positioning and alignment, visualization of the femoral and tibial cortices, recognition of anisotropy-related image distortion, and standardized MME measurement procedures under both WB and NWB conditions. Students then participated in a brief hands-on practice session prior to data collection. No formal standardized teaching script was used, and only limited real-time corrective feedback was provided during scanning practice.

All the knee and examiner participants provided written informed consent. This study was approved by the Institutional Ethics Committee of the affiliated institution (Approval No. 24-09).

**Figure 1:**
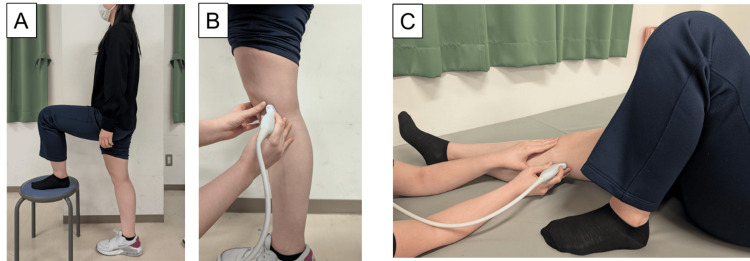
Measurement positions for MME. (A) WB position: the participant stands on the right leg with the left knee flexed on a chair for stability. (B) Probe placement in the WB condition. (C) NWB position: the participant is in a long-sitting position with the hip and knee flexed. MME: medial meniscus extrusion; NWB: non-weight-bearing; WB: weight-bearing

**Figure 2:**
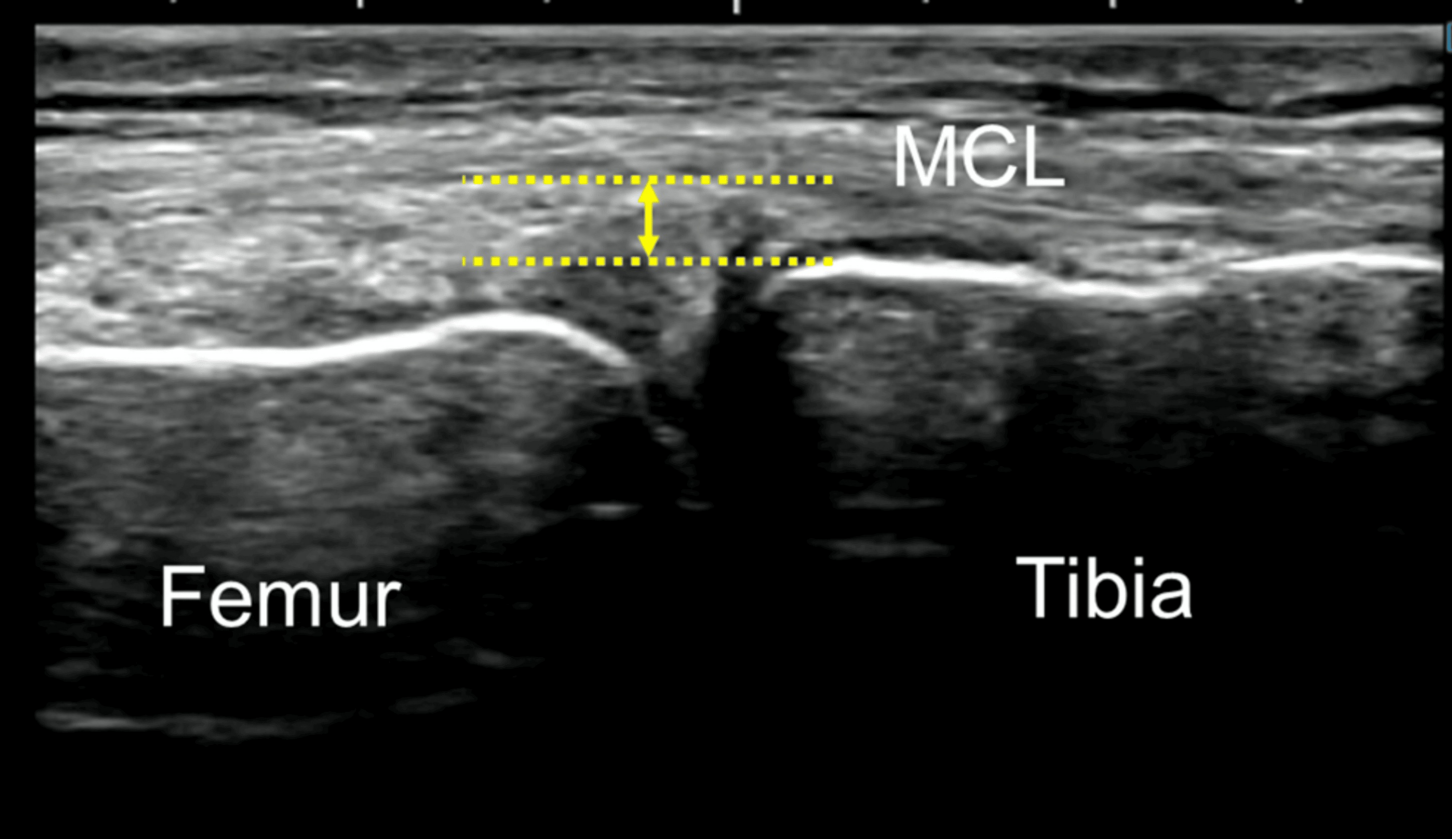
MME measurement method. The distance from the line connecting the cortical surfaces of the femur and tibia to the outermost edge of the medial meniscus (yellow arrow). MME: medial meniscus extrusion; MCL: medial collateral ligament

Measurements were obtained using an ultrasound system (SNiBLE yb, Konica Minolta, Tokyo, Japan) with a linear probe (L11-3, Konica Minolta). MME was measured by placing the probe parallel to the medial collateral ligament, using a line connecting the medial femoral condyle and the center of the medial joint space as a reference identified via palpation. Measurements were taken of the right knee in full extension under WB and NWB conditions. For WB, the knee participants stood on the right leg with the left knee flexed on a chair (Figures 1A, 1B). For NWB, the knee participants were in a long-sitting position with the left knee flexed (Figure 1C). Three consecutive measurements were performed; the probe was removed and repositioned between scans. The order of the examiner participants was randomized. To ensure blinding, all MME distances were measured from the saved images by an independent PT (>5 years of ultrasound experience, 15 years clinical experience) using ImageJ software (version 1.54g; National Institutes of Health, Bethesda, MD, USA) [13]. MME was defined as the distance from the line connecting the cortical margins of the femur and tibia to the outermost edge of the meniscus (Figure 2).

Statistical analyses were performed using Modified R Commander 4.3.3 (CRAN, Freeware) for Windows [14]. Intra-rater reliability was assessed using ICC(1, 1) for the second and third measurements. Inter-rater reliability was assessed using ICC(2, 1) based on the mean of the three trials. A Bland-Altman analysis was used to detect systematic errors [15]. If a systematic error was present, the limits of agreement (LOA) were calculated; otherwise, the minimal detectable change at the 95% confidence level (MDC95) was determined. The significance level was set at 5%.

## Results

Intra-rater reliability (ICC(1, 1)) ranged from 0.693 to 0.705 for the students; for the PT, it was 0.799 for the WB and 0.774 for the NWB (Table 1). The Bland-Altman analysis revealed a proportional bias only in Student A’s NWB measurements; no other conditions showed fixed or proportional biases. The MDC95 ranged from 0.34 to 0.50 mm for the students; for the PT, it was 0.31 mm for the WB and 0.35 mm for the NWB (Table 2).

**Table 1:** MME measurement values and intra-rater reliability (ICC(1, 1)) for each examiner. Unit: mm is used for all MME and SEM values. Values for MME are presented as mean (standard deviation). CI: confidence interval; ICC: intraclass correlation coefficient; MME: medial meniscus extrusion; NWB: non-weight-bearing (long-sitting position); PT: physical therapist; SEM: standard error of measurement; Students A and B: third-year students; WB: weight-bearing (single-leg weight-bearing in a standing position)

WB or NWB	Examiner	MME			Relative reliability		SEM
		Trial 1	Trial 2	Trial 3	ICC(1, 1)	95% CI	
WB	Student A	0.91 (0.18)	0.96 (0.17)	0.94 (0.24)	0.699	0.329-0.921	0.01
	Student B	1.14 (0.15)	1.15 (0.37)	1.14 (0.28)	0.693	0.321-0.920	0.02
	PT	1.01 (0.28)	1.00 (0.28)	1.08 (0.33)	0.799	0.500-0.950	0.01
NWB	Student A	0.80 (0.25)	0.79 (0.17)	0.93 (0.34)	0.705	0.338-0.923	0.01
	Student B	0.94 (0.25)	0.96 (0.29)	1.11 (0.40)	0.700	0.331-0.922	0.02
	PT	0.95 (0.17)	0.91 (0.18)	0.93 (0.26)	0.774	0.455-0.944	0.01

**Table 2:** Bland-Altman analysis for intra-rater reliability. Unit: mm is used for all MDC95 and LOA values. CI: confidence interval; LOA: limit of agreement; MDC95: minimal detectable change at 95% confidence level; NWB: non-weight-bearing (long-sitting position); PT: physical therapist; Students A and B: third-year students; WB: weight-bearing (single-leg weight-bearing in a standing position) p < 0.05 was considered statistically significant. Significant values are indicated by an asterisk (*).

WB or NWB	Examiner	Fixed bias		Proportional bias			LOA	MDC95
		95% CI	p-value	Slope	95% CI	p-value		
WB	Student A	−0.12 to 0.17	0.699	−0.37	−1.14 to 0.39	0.279	-	0.34
	Student B	−0.17 to 0.20	0.879	0.30	−0.30 to 0.91	0.266	-	0.44
	PT	−0.21 to 0.06	0.222	−0.16	−0.63 to 0.30	0.421	-	0.31
NWB	Student A	−0.32 to 0.05	0.120	−0.70	−1.15 to −0.26	0.008*	−0.57 to 0.29	-
	Student B	−0.36 to 0.06	0.142	−0.35	−0.99 to 0.29	0.226	-	0.50
	PT	−0.16 to 0.14	0.851	−0.43	−1.13 to 0.28	0.189	-	0.35

For inter-rater reliability (ICC(2, 1)), agreement between Student B and the PT for the WB condition was 0.895. In contrast, the ICC(2, 1) was 0.204 between Student A and the PT, and 0.194 between the two students. The Bland-Altman analysis identified a significant fixed bias between Student B and the PT for the WB condition, although the LOA was narrow at 0.07-0.15 mm. The MDC95 for other inter-rater comparisons ranged from 0.40 to 0.65 mm (Table 3).

**Table 3:** Inter-rater reliability (ICC(2, 1)) and Bland-Altman analysis. Unit: mm is used for all SEM, MDC95, and LOA values. CI: confidence interval; ICC: intraclass correlation coefficient; LOA: limit of agreement; MDC95: minimal detectable change at 95% confidence level; NWB: non-weight-bearing (long-sitting position); PT: physical therapist; SEM: standard error of measurement; Students A and B: third-year students; WB: weight-bearing (single-leg weight-bearing in a standing position) p < 0.05 was considered statistically significant. Significant values are indicated by an asterisk (*).

WB or NWB	Comparison	Relative reliability		SEM	Bland-Altman analysis					LOA	MDC95
		ICC(2, 1)	95% CI		Fixed bias 95% CI	p-value	Slope	Proportional bias 95% CI	p-value		
WB	Student A vs. Student B	0.194	−0.292 to 0.720	0.20	−0.44 to 0.03	0.080	−0.51	−1.92 to 0.89	0.420	-	0.55
	Student A vs. PT	0.204	−0.561 to 0.769	0.22	−0.35 to 0.17	0.440	−0.71	−2.14 to 0.71	0.281	-	0.62
	Student B vs. PT	0.895	0.072 to 0.982	0.06	0.05 to 0.18	0.005*	−0.12	−0.37 to 0.12	0.280	0.07 to 0.15	-
NWB	Student A vs. Student B	0.405	−0.226 to 0.834	0.21	−0.41 to 0.09	0.169	−0.28	−1.45 to 0.89	0.591	-	0.59
	Student A vs. PT	0.589	−0.052 to 0.899	0.14	−0.26 to 0.08	0.258	0.28	−0.64 to 1.20	0.494	-	0.40
	Student B vs. PT	0.22	−0.591 to 0.780	0.23	−0.20 to 0.35	0.548	0.68	−0.74 to 2.10	0.301	-	0.65

## Discussion

This pilot study investigated the educational impact of a brief, 30-minute training session on the reliability of MME measurement among novices compared to an experienced PT. According to the criteria proposed by Koo and Li [16], ICC values are interpreted as follows: values < 0.5 indicate poor reliability, between 0.5 and 0.75 indicate moderate reliability, between 0.75 and 0.9 indicate good reliability, and >0.90 indicate excellent reliability. Based on these benchmarks, our results suggested that short-term education may contribute to moderate intra-rater reliability in novices; however, it did not ensure stable inter-rater proficiency across different individuals and loading conditions.

The most notable finding was the inconsistency, or reversal, of inter-rater reliability between the two students. For the WB condition, Student B achieved good agreement with the PT (ICC: 0.895), whereas Student A showed poor agreement (ICC: 0.204). Conversely, for the NWB condition, Student A reached moderate reliability (ICC: 0.589), whereas Student B dropped to poor reliability (ICC: 0.220). This variability suggests that novice skills acquired through brief training are highly volatile. These findings suggest that brief theoretical training alone may be insufficient to achieve stable and transferable ultrasound measurement proficiency among novices. The presence of proportional bias in Student A’s intra-rater measurements further indicates that without continuous feedback, novices may develop idiosyncratic habits in probe manipulation that compromise data consistency.

A primary technical hurdle contributing to measurement inconsistency is the phenomenon of anisotropy [10,17,18]. Ultrasound imaging of the meniscus is highly sensitive to the probe angle; even a slight tilt can obscure the anatomical landmarks of the femoral and tibial cortices, leading to measurement errors [10,18]. Given that MME values in healthy young adults are minimal (approximately 1 mm), a deviation of only 0.5 mm, likely caused by inconsistent probe orientation, can drastically lower ICC values. The students’ inability to maintain a strictly perpendicular angle to the joint line likely explains the wide range of inter-rater reliability observed. Furthermore, because MME values in healthy individuals are often sub-millimeter in scale, the observed measurement error may exceed clinically meaningful thresholds in some cases. This finding suggests that even relatively small inconsistencies in probe handling could substantially affect the clinical interpretability of MME measurements obtained by novices. Importantly, anisotropy may function not only as a technical challenge but also as a systematic source of measurement bias affecting both reliability and validity. These findings suggest that rapid ultrasound training without supervised probe correction may be insufficient to ensure accurate and reproducible MME assessment among novices.

Furthermore, the intra-rater reliability of the experienced PT in this study (ICC: 0.774-0.799) was classified as good, which is slightly lower than excellent reliability (ICC > 0.90) reported in previous studies such as the one by Martinese et al. [8]. This discrepancy may be explained by the restriction of range phenomenon [19]. Although previous studies often included patients with knee osteoarthritis exhibiting significant MME (3 mm or more) [3,8], our study involved healthy participants with very small MME values. In a population with such a narrow range of measurement values, any minor absolute error has a disproportionately large impact on the ICC, which is a ratio-based statistic. Therefore, the PT’s reliability should be considered clinically acceptable given the technical difficulty of measuring sub-millimeter differences in a healthy cohort.

This study has a few limitations. First, the small sample size (n = 8) limits the generalizability of the findings. Additionally, the small sample size may have contributed to instability in the ICC estimates and the wide confidence intervals observed in several analyses. Because ICC is sensitive to between-subject variability, reliability classifications such as “moderate” or “good” should be interpreted cautiously in this pilot study. Second, the training intervention lacked a hands-on feedback component, which is likely essential for overcoming anisotropy. Future research should involve larger cohorts, including patients with varying degrees of knee osteoarthritis, and evaluate students’ long-term retention of these measurement skills after more intensive practical training. Future educational interventions may benefit from supervised real-time feedback techniques, such as probe angle correction or toggle maneuvers, to improve beam alignment during MME assessment.

## Conclusions

In conclusion, this pilot study demonstrated that a brief, 30-minute educational session provided novice examiners with an initial understanding necessary to achieve moderate intra-rater reliability for MME measurements. However, the high variability in inter-rater reliability across different loading conditions and between individual students suggests that such minimal training alone is insufficient to ensure consistent clinical proficiency. The findings indicate that theoretical instruction is a valuable starting point; however, the technical nuances of ultrasound, particularly anisotropy management, likely require more intensive, hands-on feedback to reduce the gap between novice and experienced performance. Integrating standardized ultrasound training into physical therapy curricula appears feasible, although educational programs should emphasize practical skill acquisition to support reliable and clinically appropriate ultrasonographic assessment.
